# Angiotensin II receptor blocker losartan exacerbates muscle damage and exhibits weak blood pressure-lowering activity in a dysferlin-null model of Limb-Girdle muscular dystrophy type 2B

**DOI:** 10.1371/journal.pone.0220903

**Published:** 2019-08-12

**Authors:** Zoe White, Nadia Milad, Arash Y. Tehrani, William Wei-Han Chen, Graham Donen, Stephanie L. Sellers, Pascal Bernatchez

**Affiliations:** 1 University of British Columbia (UBC) Department of Anesthesiology, Pharmacology & Therapeutics, Vancouver, Canada; 2 UBC Centre for Heart Lung Innovation & St. Paul’s Hospital, Vancouver, Canada; University of Minnesota Medical School, UNITED STATES

## Abstract

There is no cure or beneficial management option for Limb-Girdle muscular dystrophy (MD) type 2B (LGMD2B). Losartan, a blood pressure (BP) lowering angiotensin II (AngII) receptor type 1 (ATR1) blocker (ARB) with unique anti-transforming growth factor-β (TGF-β) properties, can protect muscles in various types of MD such as Duchenne MD, suggesting a potential benefit for LGMD2B patients. Herein, we show in a mild, dysferlin-null mouse model of LGMD2B that losartan increased quadriceps muscle fibrosis (142%; P<0.0001). In a severe, atherogenic diet-fed model of LGMD2B recently described by our group, losartan further exacerbated dysferlin-null mouse muscle wasting in quadriceps and triceps brachii, two muscles typically affected by LGMD2B, by 40% and 51%, respectively (P<0.05). Lower TGF-β signalling was not observed with losartan, therefore plasma levels of atherogenic lipids known to aggravate LGMD2B severity were investigated. We report that losartan increased both plasma triglycerides and cholesterol concentrations in dysferlin-null mice. Other protective properties of losartan, such as increased nitric oxide release and BP lowering, were also reduced in the absence of dysferlin expression. Our data suggest that LGMD2B patients may show some resistance to the primary BP-lowering effects of losartan along with accelerated muscle wasting and dyslipidemia. Hence, we urge caution on the use of ARBs in this population as their ATR1 pathway may be dysfunctional.

## Introduction

Limb-girdle muscular dystrophy (MD) type 2B (LGMD2B) and Miyoshi myopathy are forms of MD caused by mutations in the dysferlin gene[1–3], a calcium-dependent sarcolemma repair and vesicle trafficking protein [[4,5] and reviewed in [6]]. In their late teens, patients with dysferlinopathies present with proximal and/or distal muscle weakness, which eventually results in a complete loss of ambulation. To facilitate the testing of therapeutics, a number of mouse strains have been identified (i.e. A/J, SJ/LJ [7]) or engineered (dysferlin-null [5]) to express abnormally low levels of dysferlin. These typically show slightly elevated plasma creatine kinase (CK) levels, a marker of muscle damage, and skeletal muscle biopsies reveal early muscle regeneration followed by moderate fibrofatty remodelling, accumulation of macrophages and T cells as well as muscle wasting, but without the dramatic loss of ambulation typically observed in patients [5,8]. A recent report from our group has shown that the mild phenotype of dysferlin-null mice can be exacerbated by increasing atherogenic, or non-HDL (high-density lipoprotein) levels of plasma cholesterol, which results in accelerated fibrofatty remodeling and gradual loss of ambulatory function similar to affected patients [9]. Currently, there is no cure for dysferlinopathies nor any effective management options to delay the inevitable loss of ambulation observed in this patient population.

Losartan is an angiotensin II (AngII) receptor type 1 (ATR1) blocker (ARB) routinely used to safely reduce blood pressure. In addition, others have reported that losartan has the unique ability to attenuate transforming growth factor beta (TGF-β) signaling, a key regulator of muscle homeostasis and regeneration [10–12]. In Duchenne MD (DMD), the cardiac and skeletal muscle wasting cascade believed to be partly regulated by TGF-β can be attenuated by losartan treatment, resulting in reduced serum CK, fibrosis, and calcification in the gastrocnemius, diaphragm and myocardium of *mdx* mouse model of DMD [13,14]. Other studies in the same model reported reduced diaphragm fibrosis, improved grip strength and improved *ex vivo* force frequency in the extensor digitorum longus (EDL) 6 months (mo) post-treatment [10]. Acute losartan treatment also protects against disuse atrophy following 21 days of hind-limb immobilisation and against cardiotoxin injury, where losartan improved muscle regeneration, time to heal as well as a number of *in vivo* functional readouts in the tibialis anterior (TA) of sarcopenic mice [15]. Furthermore, therapeutic benefits following muscle injury include improved muscle regeneration (4 and 18 days post-injury) and reduced fibrotic tissue formation in adult skeletal muscles [10]. In another model of MD, α2 laminin-deficient congenital MD type 1A (MDC1A) was improved with losartan treatment [16] and this tissue protection was also observed in patients [17]. Since LGMD2B patients also suffer from chronic muscle wasting and have no management options, we hypothesized that losartan could protect muscle tissues in dysferlin-deficient animals. For this purpose, the effect of chronic losartan treatment (9 mo) on muscle wasting was evaluated in two mouse models of diet-accelerated dysferlinopathy; 1) dysferlin-null mice fed a control diet, and 2) dysferlin-null mice fed a high-fat Western diet (HFD), previously shown to exacerbate LGMD2B severity [9,18]. We report that losartan treatment unexpectedly aggravated muscle wasting and fibrofatty infiltration in mild and severely dysferlinopathic mice, which correlated with a losartan-dependent atherogenic shift in their plasma lipid profile. In addition, dysferlin-null mice were resistant to the primary blood pressure (BP)-lowering effects of losartan, which was in stark contrast to WT controls. Hence our data suggest that use of losartan, and perhaps ARBs in general, should be avoided in dysferlinopathic patients and further highlights the unique pathogenesis of dysferlinopathies compared to other types of MD.

## Materials & methods

### Animal models

Mice were housed in a 12-hour/12-hour light/dark cycle, temperature-regulated facility. All animal procedures were prospectively approved by the UBC Animal Care Committee (protocol A18-0172). Experimental mice were bred using dysferlin-null mice (C57BL/6 background: *Dysf*^*tm1Kcam*^) provided by the Dr. Kevin Campbell lab [19]. Ear-clip DNA was extracted using DNeasy extraction kit (Qiagen, #69506) following manufacturer’s instructions. Mice were genotyped using a previously described dysferlin PCR protocol [20]. Euthanasia was performed under anaesthesia (3.5% v/v isoflurane, 2L O_2_) and either cervical dislocation or cardiac puncture for perfusion with warm Krebs solution as described [9,18].

### Losartan treatment

Mice were fed either a high-fat (HFD) (Harlan, TD88137; 42% kcal from fat and 34% sucrose by weight + 0.2% total cholesterol) or normal control diet (Chow; LabDiet #5001); 13% kcal from fat; 3.7% sucrose by weight) from 2 to 11 mo of age. For chronic treatment, Losartan (0.6g/L) was administered in drinking water *ad libitum* for the same duration at a dose previously shown to have biological availability and therapeutic efficacy in mouse models of DMD and connective tissue disease [10,15,21]. For acute treatment, WT and dysferlin-null mice were supplemented with the same dose of losartan (0.6g/L) *ad libitum* from 6 to 12 weeks of age. Mice provided with standard drinking water served as controls. For dose response experiments, both WT and dysferlin-null mice were treated with 6 increasing dosages of losartan spanning 6 days (0, 3.6, 7.1, 10.7, 17.8, 21.4 and 100mg/kg/d).

### Blood pressure (BP) measurements

Systemic BP was noninvasively measured using the tail cuff system (Kent Scientific CODA2). Briefly, mice were lightly anesthetised (0.75% v/v isoflurane, 1.5L O_2_) and placed on a warming tray with the tail inserted into an inflatable cuff where systolic BP (SBP) and diastolic BP (DBP) were measured. Mean arterial pressure (MAP) was calculated as follows: 1/3 x SBP + 2/3 x DBP.

### Analysis of plasma cholesterol, triglycerides and creatine kinase

Plasma was collected in heparinized tubes via cardiac puncture of mice at 11mo, centrifuged at 4,000 RPM for 10 min at 4°C and stored at -80°C. The Siemans Advia 1800 system was used to quantify plasma concentrations of creatine kinase (CK), total cholesterol (TC), high density lipoprotein (HDL-C), low density lipoprotein (LDL-C) and triglyceride (TGs) levels (assays all from Siemens) were performed according to instructions from the manufacturer and as previously published [9,18].

### Tissue processing

Muscles were fixed in 10% formalin for 24h then transferred to 70% EtOH, paraffin-embedded, sectioned to 8μm and stained with Masson’s trichrome as previously reported [9]), as frozen sectioning causes fat smearing when numerous adipocytes are present. Fat was quantified by manually tracing adipocyte containing regions (previously confirmed by perilipin staining [9]) using Aperio ImageScope. Muscle damage, which included areas of bulk inflammation and necrotic muscle fibres were also quantified as previously described [9]. Area values for each parameter were divided by total muscle area (μm^2^) to obtain percentage values. Collagen content/fibrosis was measured using a positive pixel count algorithm in Aperio ImageScope software using the following parameters: hue value of 0.66 and hue width of 0.25 and standardized to the total area of outlined sections (μm^2^). For cross-sectional area (CSA) measures, quadriceps (rectus femoris) and triceps brachii muscles stained with Masson’s were portioned into nine equal quadrants and the CSA of 100 myofibers were measured in each quadrant, totalling 900 myofibers for each muscle section. Myofiber number and the percentage of total myofibres with a displaced and/or central nuclei were counted on entire muscle cross sections using Aperio ImageScope.

### Phospho-SMAD2 immunofluorescence

As described in [22], 8μm frozen sections were placed in ice-cold EtOH for 10 min. Sections were blocked in 10% FBS, 0.03% Triton-X 100 in PBS before being incubated overnight at 4°C in 1% FBS, 0.03% Triton-X 100 in PBS (1:200; Phospho-SMAD2(Ser465/467); Invitrogen; #44–244). Sections were washed 3 x 15min in 0.03% Triton-X 100 in PBS on ice and incubated for 3h at 4°C in 1% FBS, 0.03% Triton-X 100 in PBS (AlexaFluor594 goat anti-rabbit #A11037 Invitrogen). Samples were again washed 3 x 15min in 0.03% Triton-X 100 in PBS on ice and mounted with DABCO mounting media with DAPI. Phospho-SMAD2 (pSMAD2) positive nuclei were manually quantified and divided by the total number of nuclei present (DAPI) across four images (each 20x magnification) using Aperio ImageScope taken across whole quadriceps muscles. No discrimination between myonuclei and nuclei from other cells types were made.

### Immunoblotting

Briefly, remaining frozen quadriceps were ground in liquid nitrogen, homogenized in ice-cold PBS, 1% NP40, 1mM EDTA buffer, with complete EDTA-free protease inhibitor and PhosSTOP phosphatase inhibitor tablets (Roche, Manheim, Germany), and centrifuged at 13,000g for 20 min at 4°C [23]. Protein was quantified with the DCA protein Assay (Bio-Rad). Samples were resolved on 4–15% SDS-PAGE TGX gels (Bio-Rad) and transferred onto nitrocellulose membranes (Bio-Rad) using the Trans-Blot Turbo Transfer System (Bio-Rad; mixed molecular weight program; 2.5A-25V-7min). Following transfer, membranes were blocked in 1% casein in TBS (Li-COR) for 1 hour at RT. Primary antibodies were diluted 1:1000 in 1% casein in TBST (0.1% Tween 20); p-Akt(Ser473) (#9271), t-Akt (#9272), p-ribosomal protein S6(Ser235/236) (#4858), p-ribosomal protein S6(Ser240/244) (#5364) t-ribosomal protein S6 (#2217), p-p44/42 MAP Kinase (Thr202/Tyr204) (ERK; #9101), p44/42 MAP Kinase (ERK; #9102), LC3B (#2775) and incubated overnight at 4 degrees. Membranes were then washed 3x5mins in TBST, incubated with goat anti-rabbit AlexaFluor700 (Invitrogen; #A-21038; 1:5000; 1% casein in TBST) for 45 mins at RT and rewashed 3x5mins in TBST before imaging with the Li-COR Odyssey scanner. The loading control GAPDH (#2118; Cell Signaling; 1:2000) was probed from all membranes following stripping in 2% SDS, 62.5mM Tris-HCL (pH 6.7), 100mM ß-mercaptoethanol at 50°C for 30 min and washed 5 x 5 min in TBST before re-blotting. Total and phosphorylated protein forms were standardized to individual GAPDH values before ratios of phosphorylated/total protein intensity was calculated. A common sample was loaded onto each gel to normalize for detection efficiencies across membranes. The prefixes “P” and “P” signify “phosphorylated” and “total” forms respectively.

### Measurement of isometric force

The descending thoracic aorta (ThA) was dissected from the thoracic cage and cleaned of fat and connective tissue in ice-cold Krebs solution [118mmol/L NaCl, 22.5mmol/L NaHCO_3_, 4mmol/L KCl, 1.2mmol/L NaH_2_PO_4_, 2mmol/L CaCl_2_, 2mmol/L MgCl_2_, 11mmol/L dextrose, 0.01mmol/L Ibuprofen]. Segments of the ThA (2mm) were mounted isometrically in a small vessel myograph (AS Danish Myotechnology, Aarhus N, Denmark), left to equilibrate for 30min at 37°C in Krebs solution aerated continuously with 95% O_2_-5% CO_2_, followed by optimal tension stretching (6.0mN) for 30min as previously described [24]. KCl (30mmol/L) and concentration-response with phenylephrine (PE) (3nM to 100μM) were performed. Contraction was calculated as the % increase or decrease in force with respect to untreated WT mice, where the maximum recorded response was set to 100%. N^ω^-nitro-L-arginine methyl ester (L-NAME, 200mM) was used to block NO release.

### PECAM immunohistochemistry

PECAM (Cell Signaling #77699; 1:100) was stained on paraffin sections cut at 4μm. Sections were deparaffinized in 2 x 10 min xylenes; 2 x 10 min 100% EtOH; 5 min 95% EtOH; 5 min 70% EtOH and dH_2_O, then washed for 3 x 5 min minutes in PBS at RT. Antigen retrieval was performed in 10mM citrate buffer until boiling for 8–15 min in the microwave and cooled at RT for 20–30 min. Sections were washed 3 x 5 min in PBS, quenched in 3% H_2_O_2_ –MeOH for 15 min at RT before being rewashed in PBS 3 x 5 min. Sections were then blocked in 3% BSA in PBS for 1h at RT. Primary antibody was diluted in 3% BSA in PBS and slides incubated overnight at 4°C. Sections were rinsed again 3 x 5 min in PBS at RT, goat anti rabbit secondary antibody (Vector Laboratories; #BA-1000; 1:350) diluted in 3% BSA in PBS and applied for 30 min at RT before washing again for 3 x 5 min in PBS. VECTASTAIN ABC reagents (Vector Laboratories; #SK6100) were pre-complexed 30 min prior to application as specified by the manufacturer and applied to sections for 30 min at RT, before rinsing sections 3 x 5 min in PBS. ImmPACT DAB reagents (Vector Laboratories; #SK-4105) was added for 1–2 min, rinsed in dH2O, counterstained with Haematoxylin and cover-slipped. Average vessel density was quantified on PECAM stained sections by averaging the number of blood vessels across 4–6 randomised images (dependent on muscle size) taken at 20x magnification using Aperio ImageScope software.

### Sudan IV and Van Geisson staining

Whole ThA aortas fixed in 10% formalin were cleaned and stained with Sudan IV. Briefly, cleaned aortas were rinsed in 70% EtOH and placed into Sudan IV (5g Sudan IV in 500mL 70% EtOH and 500mL acetone) for 20 min. Aortas were rinsed briefly in 80% EtOH, soaked in another exchange of 80% EtOH for 20 min washed under running tap water for 60 min and then stored in 10% formalin until imaging. An apolipoprotein E deficient mouse aorta (a common model of atherosclerosis) served as a positive control. Images were taken with a through a Zeiss KL2500-LCD dissecting microscope (Diagnostic Instruments) with a Samsung A8 camera. Remnant ascending aortas were embedded cross-sectionally, stained with Van Geisson using standard method, and representative images taken at 20x magnification using Aperio ImageScope software.

### Statistical analyses and data availability

Statistical analyses were performed using GraphPad Prism 6. One-way analysis of variance (ANOVA) was used to compare the means of each group and Fisher’s post-hoc tests of least significant difference used to analyse direct mean comparisons unless stated otherwise. A p-value of less than 0.05 was considered statistically significant. Figures show data as mean plus standard error of the mean (SEM).

## Results

### Losartan treatment exacerbates muscle wasting in dysferlin-null mice

**Fig 1:**
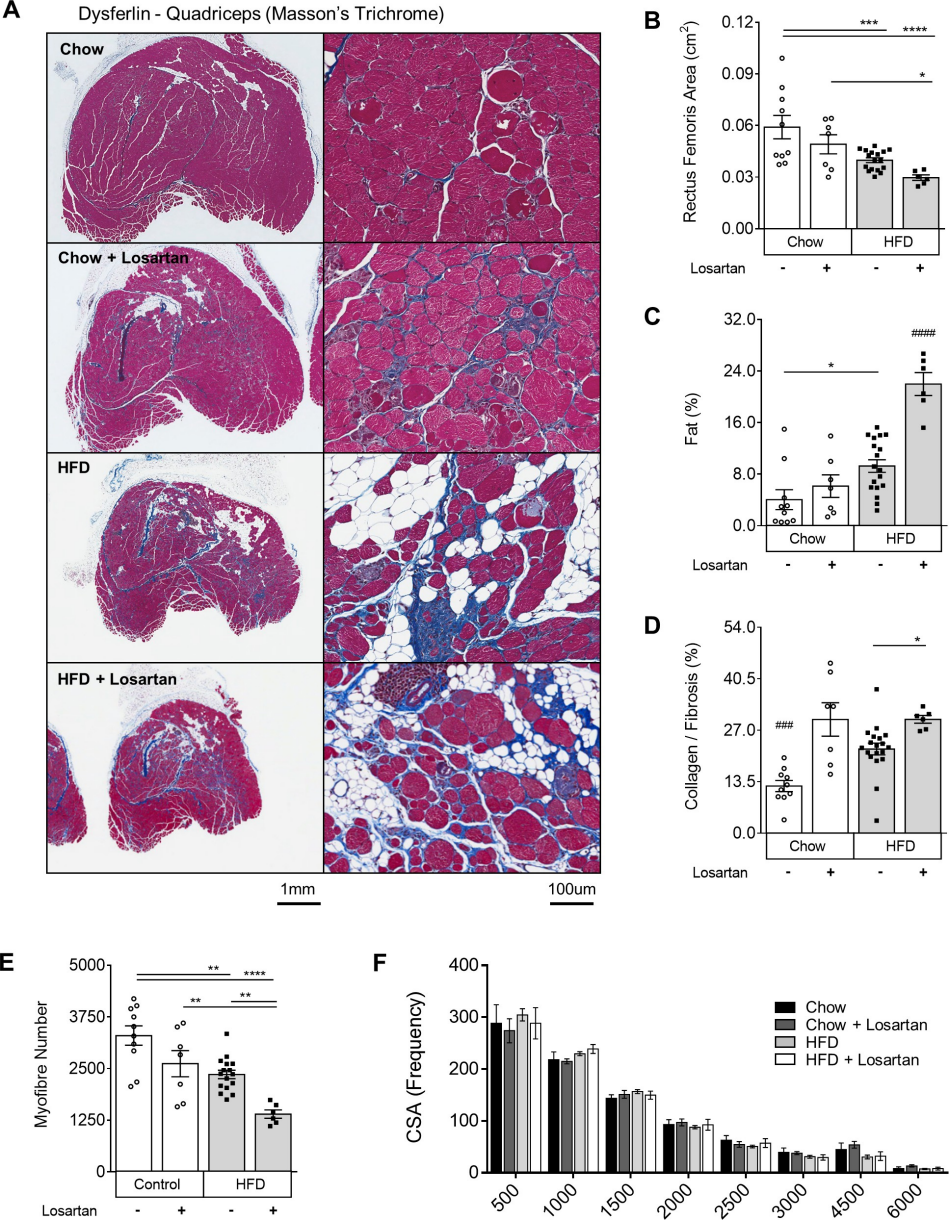
Losartan treatment aggravates quadriceps muscle damage in chow- and HFD-fed dysferlin-null mice. Representative images of quadriceps from mice after Masson’s trichrome staining (A) and quantification of total rectus femoris area (B), percentage area of fat (C), percentage area of fibrosis/collagen infiltration (D), total myofibre number (E) and cross-sectional area (CSA) frequencies (F). Mean±SEM; P<0.05 (*), P<0.01 (**), P<0.001 (***), P<0.0001 (****), One-way ANOVA with Fisher’s post-hoc tests of least significant differences. Hash (#) indicates significantly different from all other groups, P<0.001 (###), P<0.0001 (####); Scale bars for 2x and 20x images are 1mm and 100μm, respectively. Muscle tissue (pink); fibrosis (blue); fat/adipocytes (white). Chow (N = 10); Chow losartan (N = 7); HFD (N = 18); HFD losartan (N = 6).

To test the therapeutic potential of losartan in LGMD2B, dysferlin-null mice were treated with 0.6g/L of losartan in drinking water for 9 mo. Staining of both quadriceps (rectus femoris) and triceps brachii muscle groups with Masson’s trichrome revealed that losartan exacerbated muscle pathology in a diet and drug-specific manner (**Figs 1 and S1)**. Losartan exerted minor detrimental effects in chow-fed mice, whereby heightened fibrosis was observed in rectus femoris (142%; P<0.001; **Fig 1A and 1D**), but not triceps brachii muscle groups (**S1A and S1D Fig**). Conversely, in HFD-fed mice losartan treatment resulted in a profound 32% decrease in rectus femoris and triceps brachii size (**Figs 1A and 1B and S1A and S1B;** P<0.05), as well as increased fat infiltration (129% and 202%; P<0.05) and collagen deposition (81% and 54%; P<0.05) in each muscle, respectively, compared to chow-treated mice (**Figs 1A, 1C and 1D and S1A, S1C and S1D**). Consistent with profound muscle atrophy, losartan caused a 40% decrease (P<0.05) in total rectus femoris myofibre number in HFD-fed mice, irrespective of changes to myofibre cross-sectional area (**Fig 1E and 1F**) and the percentage of centrally nucleated myofibers (**S2A Fig**). In triceps brachii however, muscle atrophy was associated with reduced myofibre number, an increased frequency of smaller myofibres (500–1000μm^2^) (**S1E and S1F Fig**), and in HFD-fed muscles increased rates of central nucleation (**S2A Fig**). Active sites of muscle necrosis and bulk inflammation in both muscle groups was minor (<10%; **S2B Fig**), yet reduced in rectus femoris muscles of HFD-fed mice. Despite obvious histological changes, plasma CK levels (a bi-phasic marker of muscle damage) were unaffected (**S2C Fig**), as published by others in dysferlin-null animals [25]. Together, these data show an exacerbation of muscle wasting by losartan in both the triceps brachii and quadriceps (rectus femoris), two muscle groups severely affected by LGMD2B [8,9].

### Losartan does not attenuate TGF-β-related signalling and shows limited effects on muscle protein synthesis (AKT/rpS6) or autophagy (LC3B) pathways

**Fig 2:**
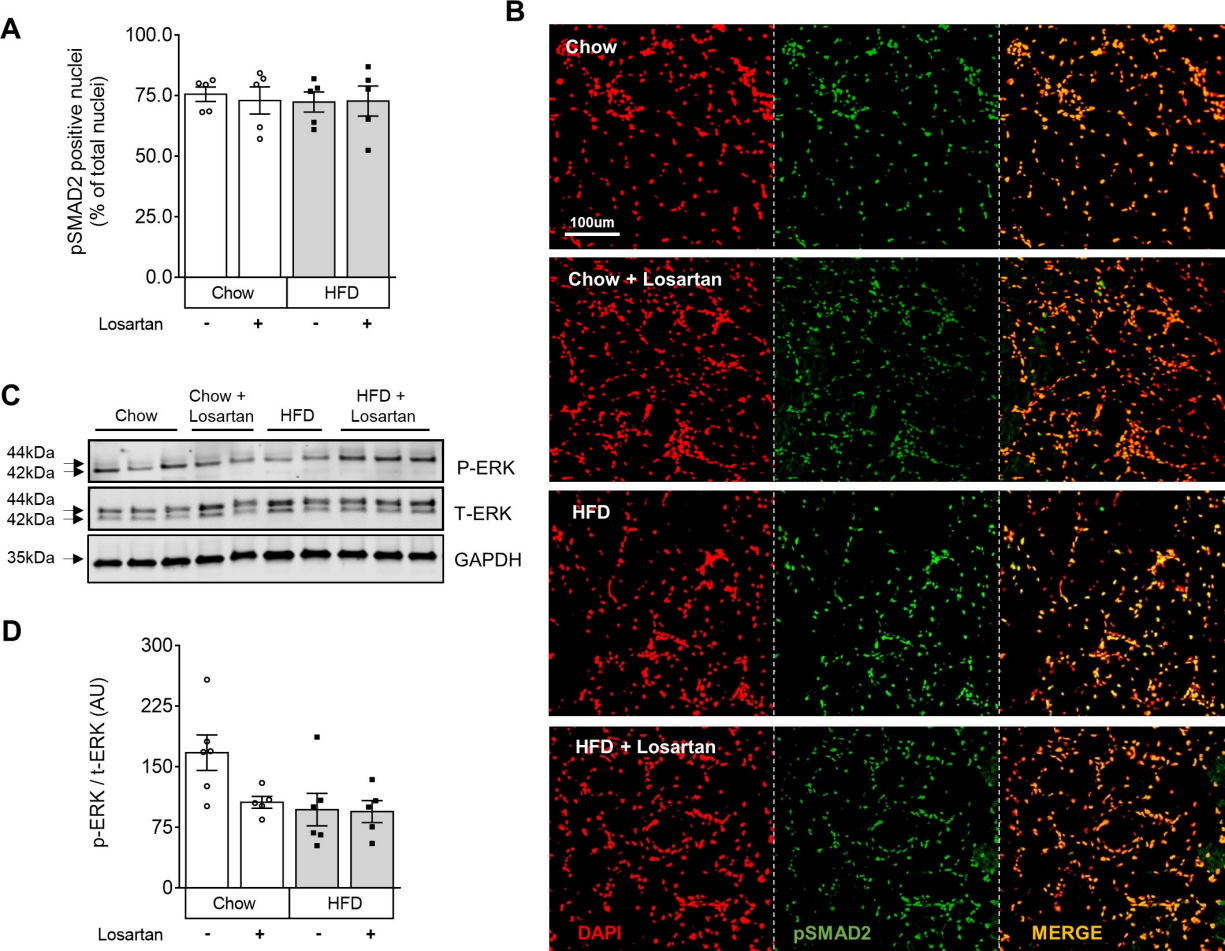
Canonical (pSMAD2) and non-canonical (p44/42 MAP Kinase; ERK-1/2) TGF-β signalling in quadriceps muscle lysates are unaffected by diet or losartan treatment. Percentage of total nuclei positive for p-SMAD2(Ser465/467) quantified on whole quadriceps muscle sections (A) with representative pSMAD positive images (B), and quantitation of p-ERK(Thr202/Tyr204) standardised to t-ERK (C,D). DAPI (Red); pSMAD2 (Green); Merge (yellow). Mean±SEM; One-way ANOVA with Fisher’s post-hoc tests of least significant differences. Y-axes represent arbitrary units (A.U.) unless stated. Scale bar for 20x images is 100μm. p-ERK and t-ERK were blotted on separate gels, and GAPDH blotted on each to control for loading. Immunoblots share the same loading order, sample concentration and loading control. Full blots were imaged separately and thus have differing exposures. Chow (N = 5–6); Chow losartan (N = 5); HFD (N = 5–6); HFD losartan (N = 5).

Since losartan is often linked to attenuated TGF-β signalling [10,16], downstream SMAD2 and ERK1/2 activation were investigated by immunohistochemistry and Western blotting (**Fig 2A–2D**). While robustly expressed, the percentage of pSMAD2 positive nuclei in whole quadriceps muscles were similar across all experimental groups (**Fig 2A and 2B**), as was the average number of DAPI positive nuclei counted across all images: Chow, 280 ± 36; Chow + Losartan; 393 ± 50; HFD, 390 ± 74; and HFD + Losartan, 410 ± 64). Protein lysates from whole quadriceps were separated by SDS-PAGE and immunoblotting revealed that p-ERK(Thr202/Tyr204) standardised to t-ERK (which reflects activation of this protein) was also unaffected by diet or losartan treatment (**Fig 2C and 2D**). Finally, a major regulator of muscle protein homeostasis, the IGF-1/insulin signalling pathway, was also studied. The activation of mTORC1 by protein kinase B (PKB)/AKT or directly by nutrients can promote protein synthesis by phosphorylating two major targets, ribosomal protein S6 kinase beta-1 (S6K1) and eukaryotic translation initiation factor 4E-binding protein 1 (4E-BP1), which can be assessed by rpS6(Ser235/236) [26]. Immunoblotting of phosphorylated AKT (Ser473; p-AKT) standardized to total AKT (t-AKT) (**S3A and S3B Fig**), although unaffected in control diet-fed muscle, was significantly reduced in HFD-fed conditions, an effect abolished by losartan treatment (**S3A and S3B Fig**). P-rpS6 standardized to t-rpS6 was less robust, and overall, unaffected by diet or losartan (**S3A and S3C Fig**). Given that mTORC1 activation is also shown to negatively regulate autophagy in skeletal muscle [27], the ratio of LC3BII/I (a marker of autophagy) was also evaluated. Consistent with a lack of rpS6 phosphorylation, ratios of LC3BII/I were also similar across all experimental groups (**S3A and S3D Fig**). Combined, these data provide evidence that losartan does not modulate TGF-β activation in mild or severe models of dysferlinopathy and that classical pathways responsible for muscle homeostasis (protein synthesis and degradation) were not impacted by losartan despite significant muscle atrophy.

### Losartan induces an atherogenic shift in plasma cholesterol and TG levels

**Fig 3:**
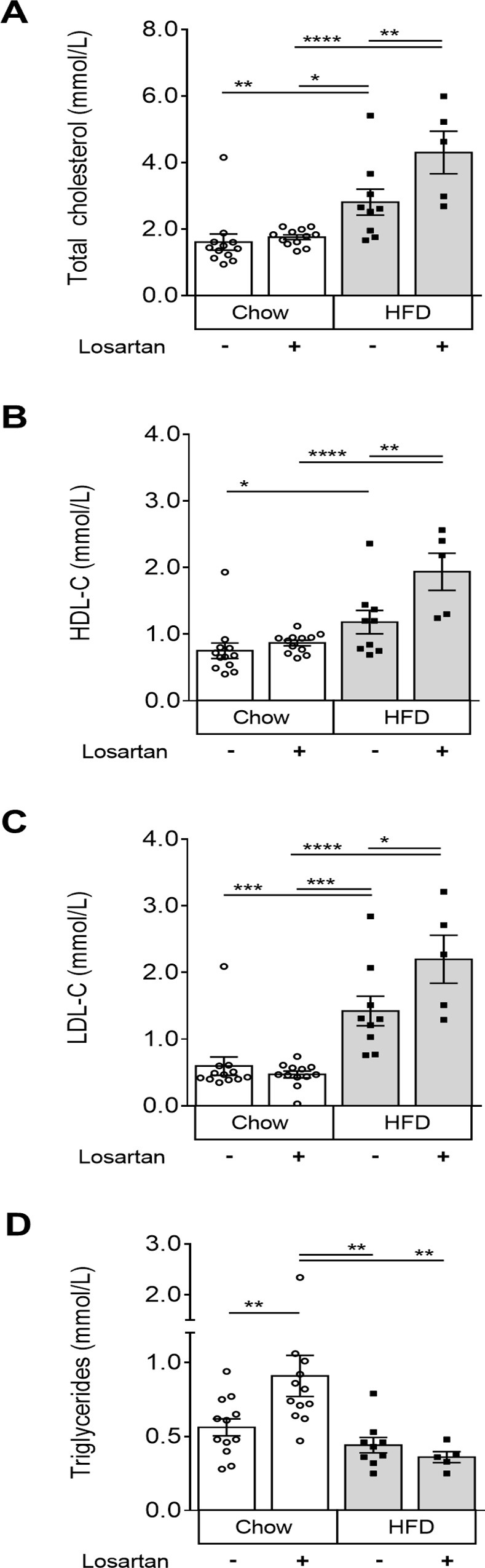
Plasma cholesterol and triglyceride levels in chow and HFD fed dysferlin-null mice with or without losartan treatment. Total cholesterol (A), high-density lipoprotein (HDL-C) (B), low-density lipoprotein (LDL-C) (C) and triglycerides (TG) (D). Mean±SEM; P<0.05 (*), P<0.01 (**), P<0.001 (***), P<0.0001 (****), One-way ANOVA with Fisher’s post-hoc tests of least significant differences. Chow (N = 12); Chow losartan (N = 12); HFD (N = 9); HFD losartan (N = 5).

In other types of MD, losartan can protect against muscle damage while reducing plasma TGs and improving the “good” (HDL) to “bad" (LDL) cholesterol ratio [28,29]. Hence, plasma lipoprotein levels were examined. We observed that under normal chow conditions, losartan increased plasma TG levels by 62.5%, leaving TC, HDL-C and LDL-C unaffected (P<0.01; **Fig 3A–3D**). Conversely, in HFD-fed animals, losartan elevated TC, HDL-C and LDL-C (P<0.05; **Fig 3A–3C**), independent of changes to circulating TG (**Fig 3D**). These data suggest that the losartan-mediated exacerbation of muscle damage may be related to a shift in atherogenic, LDL-C or TG lipoprotein components.

### Dysferlinopathic mice are non-responsive to the endothelial function-activating and BP-lowering effects of losartan

**Fig 4:**
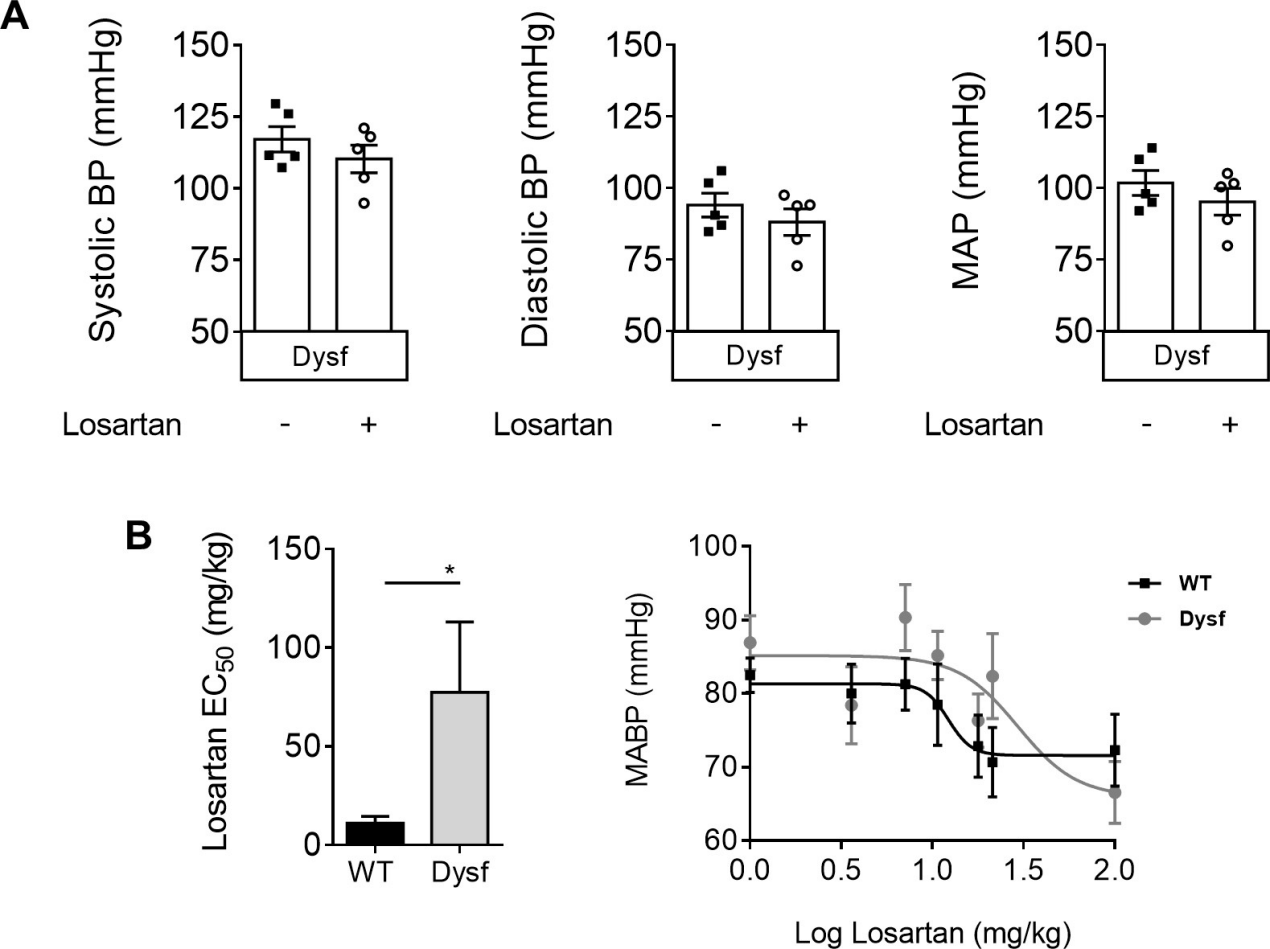
Dysferlin-null mice exhibit reduced sensitivity to the blood pressure (BP)-lowering effects of losartan. Systolic, diastolic and mean arterial blood pressure measurements (MABP) in dysferlin-null mice treated acutely (6 weeks) with a 0.6g/L dose of losartan (A), and EC_50_ of losartan dose response curves in both WT and dysferlin-null cohorts (B). Mean+SEM; P<0.05 (*), unpaired student t-test (two-tailed). N = 5–7 mice per group.

**Fig 5:**
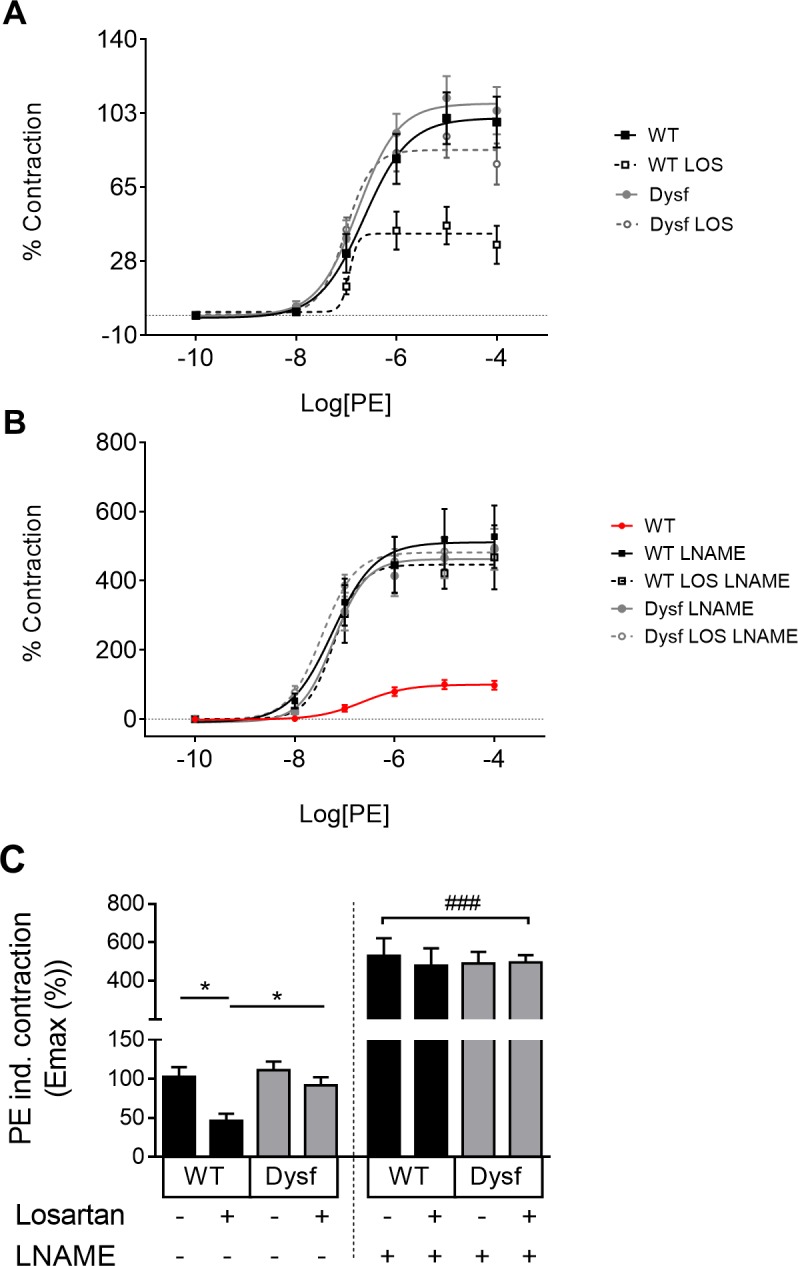
Dysferlinopathic mice are non-responsive to the NO-potentiating effects of losartan. Concentration-response curves of PE contraction pre (A) and post (B) LNAME (100uM) in thoracic aorta sections following losartan treatment. EC_50_ values are presented in bar graph (C). Contraction is expressed as a percentage of untreated WT values. Data are +SEM; P<0.05 (*). Hash (###) indicates significantly different from non LNAME treated vessels, P<0.001; One-way ANOVA with Tukey’s Post Hoc tests. N = 5–8 mice per group.

The effect of losartan on dysferlin-null muscle prompted us to test its primary effect on BP in two additional cohorts of dysferlin-null mice (**Fig 4**). Six weeks of losartan treatment failed to lower systolic, diastolic and mean arterial blood pressure (MABP) in dysferlin-null mice, compared to untreated controls (**Fig 4A**). Dose-response analyses revealed a right shift in MAP sensitivity in response to losartan as quantified by EC_50_ in dysferlin-null compared to WT mice (**Fig 4B;** P<0.05). Having recently shown that losartan can improve endothelial function via release of the vasodilatory mediator, nitric oxide (NO) [21], *ex vivo* myography was used to test the effect of acute losartan treatment on vascular NO release. While aortic rings from WT mice displayed reduced contractility in response to losartan (an effect fully reversed using the NO synthase inhibitor L-NAME) (**Fig 5A and 5B**), PE-induced contractility was unaffected in dysferlin-null vessels. These results confirm severe abnormalities in vascular function in response to losartan in dysferlin-null vessels.

### Losartan increases vascular density but has no effect on atherosclerotic burden nor aortic morphology in dysferlin-null tissues

To visualize the chronic effects of losartan on vascular pathology and angiogenic capacity, vessel density was quantified using PECAM1/CD31 stained muscle cross-sections (**S4A–S4C Fig**). In settings of increased muscle damage and fibrosis we observed increased vascular density in triceps brachii **(S4A and S4B Fig)** and rectus femoris (**S4C Fig;** quantification only) following HFD and/or Losartan treatment. Despite the presence of elevated lipids, HFD-Losartan treated aortas displayed no sign of atherosclerosis as measured by Sudan IV staining (**S4D Fig**) and no overt structural changes to vessel morphology were observed on representative Van Geisson stained ascending aortic segments (**S4E Fig**).

## Discussion

The current study provides evidence in a preclinical model of dysferlinopathy that losartan does not prevent, but rather exacerbates muscle wasting and damage, which is in stark contrast to its protective effects in other types of MD and muscle disease. Previous work by us and other groups have shown that losartan can ameliorate the manifestation of muscle disease in mouse models of DMD, MDC1A, Marfan syndrome and sarcopenia, likely by attenuating the pro-fibrotic action of TGF-β signalling [10,13,16,30,31]. This is in addition to studies that have documented prophylactic effects when targeting the same pathways using angiotensin converting enzyme inhibitors [17,32]. Rationalization of these unexpected observations may be found in a report that glucocorticosteroids used to treat other forms of MD are not effective in dysferlinopathic patients [33], indicating that loss of dysferlin causes unique and distinctive changes in muscle signalling and homeostasis, compared to other MDs.

### Losartan and TGF-β signalling in MD

The unexpected results observed in the current study argue against a therapeutic, TGF-β-attenuating role for losartan in dysferlinopathies. Elevated TGF-β signalling has been documented in DMD patients, congenital MDC1A, as well as both *mdx* mouse and golden retriever dog models of DMD [10,16,34,35]. Since TGF-β overexpression can inhibit satellite cell activation, and in injured muscle promote the differentiation of myogenic progenitors into fibrotic cells [12,16,36], TGF-β elevation in DMD likely plays an active part in the promotion of profibrotic remodelling [16]. In a recent study, L-158809 (an analogue of losartan) reduced muscle fibrosis and inflammation in MDC1A dy^w^/dy^w^ mice and improved ambulation via reduced TGF-β and pSMAD2/3 signalling [16]. In sarcopenic muscle, losartan was also able to rescue necessary Pax7 and myogenic (MyoD and MyoG) signalling interactions essential for muscle regeneration, in a TGF-β-driven, Smad2/3 and MAPK-dependent manner [15,37]. Similar therapeutic properties also restored regenerative capacity in both DMD and MDC1A mouse models following toxin-induced muscle damage [10,16]. In the current study, despite documented TGF-β upregulation in dysferlin-deficient tissues [38,39], we did not detect inhibition of SMAD2 or ERK phosphorylation following chronic losartan treatment, which suggests that loss of dysferlin may impair losartan signalling (see below). If this assessment is correct, the contrasting effects of losartan in relatively similar genetic models of muscle disease (DMD and LGMD2B) suggests that caution must be taken when testing experimental therapies in animal models harbouring mutations of the dystrophin-associated glycoprotein complex. For instance, the common use of mdx/utrophin double mutant mice as a more severe alternative to the mild mdx model of DMD may result in misleading outcomes if used to test therapies, as the probability of patients exhibiting double DGC mutations is extremely low. Instead, our team has shown that modulating atherogenic plasma lipoprotein levels, particularly LDL-C and TG, exacerbates muscle wasting to levels closer to what is generally seen in patients [9] which could be beneficial for testing of advanced therapies.

### Losartan aggravates plasma lipid abnormalities but not CK in dysferlinopathies

We have recently demonstrated that both dysferlin-null and *mdx* mice exhibit drastically worsened muscle pathology and intramuscular fat accretion when plasma lipoprotein levels are elevated to an atherogenic, LDL and TG-rich state [9,18], thus emphasising a strong relationship between lipids and muscle homeostasis in multiple forms of MD. In particular, abnormal plasma TG, phospholipids, free cholesterol, cholesterol esters and total cholesterol concentrations in DMD patients [40], as well as significant lipid accumulation in dysferlin-deficient myofibers, can be observed prior to the replacement of muscle area with adipocytes [8]. This suggests either an inherent lipid handling defect or inefficient membrane repair in response to lipotoxicity. Indeed, changes influencing phospholipid composition and oxidation can significantly alter sarcolemmal stability and calcium signalling, as observed in a number of other muscle wasting [41–44]. In general, ARBs (including losartan) as well as other anti-hypertensives are shown to improve plasma lipid profiles and/or lipoprotein composition in a number of clinical and pre-clinical disease models [13,28,45–49]. Moreover, losartan is known to reduce muscle fibrosis concurrent with increased HDL-C concentration, and reduced TG and TC in *mdx* mice [13]. Very few studies have explored the mechanisms behind losartan’s lipid-lowering effects, however other ARBs including Telmisartan and Irbesartan can modulate peroxisome proliferator-activated receptor gamma (PPAR-γ) agonist activity, a key regulator of lipid metabolism, while others have suggested high lipophilicity as a possible mediator [50]. Whether these effects and those reported herein are due to losartan’s anti-ATR1 properties or its significant off-target effects [21] is unknown. The extent to which these pathways are modified in dysferlin-null tissue warrants further investigation, although studies have reported aberrant expression of very-low-density lipoprotein receptor, reduced LDL receptor-associated protein and reduced uptake of cholesterol containing particles in dysferlin-null muscle [38]. Elevated expression of the lipogenic marker CCAAT/enhancer binding protein- δ, has also been reported in dysferlin-null A/J and BLAJ skeletal muscles prior to disease onset (3 mo), suggesting an early induction of signalling pathways that promote an adipogenic lineage [8].

Based on the dramatic changes in muscle size in losartan-treated animals, one can anticipate changes in muscle weights; however plasma CK (a commonly used marker of muscle damage in both clinical and preclinical muscle disease) was not affected in the present study, as shown by others [25]. In *mdx* mice, reduced plasma CK and improved lipid profiles (reduced TG, increased HDL-C) were correlated with reduced muscle damage and pathology following chronic losartan treatment [13]. Interestingly, in our study plasma CK concentrations did not correlate with muscle damage in dysferlin-null mice; a phenomenon that has been noted in late stage disease in both *mdx* [51] and DMD patients [reviewed in [52]], where CK levels can vary dramatically over time, and with the loss of viable myofiber area [51–53].

### Anti-hypertensive treatments in LGMD2B

How blood pressure lowering medications impact both vascular and muscle tissue homeostasis in the LGMD2B patient population is poorly understood. Although, previous work has shown that Diltiazem, a Ca^2+^ channel blocker with blood pressure lowering capabilities, can elicit partial protection in dysferlin-null tissues using acute damage models [54]. Our group has recently shown that losartan mediates its off-target, NO-protective effects on vascular tissues in a VEGFR-dependent manner [21]; a function shown to be ablated in dysferlin-deficient cells [55]. Moreover, losartan has been shown to block AngII induced, Ca^2+^-regulated lysosome fusion and lipid raft formation [56], which may have implications in dysferlin-deficient cells, given their already inherent loss of Ca^2+^-regulated membrane repair [5]. While a growing number of studies report profound vascular abnormalities in MD [9,18,55,57–59], why dysferlin-null tissues also display aberrant signalling in response to the blood pressure-lowering effects of ATR-1 blockade is unknown. Significant downregulation of ATR-1 and ATR-1 associated proteins has however been documented in dysferlin-null heart tissues [60], which supports the notion that the loss of dysferlin may have an impact on losartan’s biological function. Whether other blood pressure-lowering drugs which do not target the AngII type 1 receptor, such as angiotensin 1 converting enzyme inhibitors or β-blockers, would be a preferable pharmacological approach to treat hypertension in this patient population should be explored.

In summary, despite the efficacy of losartan in ameliorating muscle pathology in other forms of MD, the current study demonstrates that in the dysferlin-null mouse model of LGMD2B, losartan exacerbates muscle wasting concurrent with an atherogenic shift in plasma lipid profiles. When combined with earlier studies detailing lipid abnormalities in many forms of MD, this report provides an early indication of plasma lipid handling defects in dysferlinopathies, which may be a primary contributor to muscle disease pathogenesis. More importantly, our data are the first to document the reduced efficacy of losartan to lower blood pressure and promote endothelial function in dysferlin null tissues. Together, these data highlight important heterogeneities between dysferlinopathies and other types of MD, and stresses the need to assess the safety and efficacy of AngII-dependent therapies in the LGMD2B patient population.

## Supporting information

S1 Fig Losartan treatment aggravates triceps brachii muscle damage in chow- and HFD-fed dysferlin-null mice. Representative images of triceps brachii from mice after Masson’s trichrome staining (A) and quantification of total triceps brachii area (B), percentage area of fat (C), percentage area of fibrosis/collagen infiltration (D), total myofibre number (E) and cross-sectional area (CSA) frequencies (F). Mean±SEM; P<0.05 (*), P<0.01 (**), P<0.001 (***), P<0.0001 (****), One-way ANOVA with Fisher’s post-hoc tests of least significant differences. Scale bars for 2x and 8x images are 1mm and 300μm, respectively. Muscle tissue (pink); fibrosis (blue); fat/adipocytes (white). Chow (N = 8); Chow losartan (N = 7); HFD (N = 8); HFD losartan (N = 6).
(TIF)

S2 Fig Muscle regeneration and plasma CK levels in chow- and HFD-fed dysferlin-null mice with or without losartan treatment. Percentage of centrally nucleated myofibres (A) and the percentage of muscle damage (B) in rectus femoris and triceps brachii muscle groups, and levels of plasma CK (C). Mean±SEM; P<0.05 (*), P<0.01 (**), P<0.001 (***), P<0.0001 (****), One-way ANOVA with Fisher’s post-hoc tests of least significant differences. Chow (N = 12); Chow losartan (N = 12); HFD (N = 9); HFD losartan (N = 5).
(TIF)

S3 Fig Anabolic (AKT/rpS6) and autophagy (LC3B) signaling in quadriceps muscles of chow- and HFD-fed dysferlin-null mice with or without losartan treatment. Quantitation of p-AKT(Ser473) standardised to t-AKT (A,B), p-rpS6(Ser235/236) to t-rpS6 (A,C) and ratio of LC3BII/I (A,D). Mean+SEM; P<0.05 (*), P<0.01 (**), P<0.001 (***). One-way ANOVA with Fisher’s post-hoc tests of least significant differences. Y-axes represent arbitrary units (A.U). p-AKT, p-rpS6 and LC3B were cut from the same gel and blotted with respective antibodies, as were t-AKT and t-rpS6. Both total and phosphorylated strips for rpS6 were stripped and blotted for GAPDH to check for loading efficiencies. Full blots were imaged separately and thus have differing exposures. Chow (N = 6); Chow losartan (N = 5); HFD (N = 6); HFD losartan (N = 5).
(TIF)

S4 Fig Vessel density, atherosclerotic burden and aortic morphology in chow- and HFD-fed dysferlin-null mice with or without losartan treatment. Representative images (triceps brachii only; A) and quantitation of vessel density for triceps brachii rectus femoris (B) and rectus femoris (C); Scale bar is 100μm. Representative images of Sudan IV stained thoracic aorta segments in ApoE (Control) and HFD Losartan treated dysferlin-null mice (D); Scale bar is 0.5cm. Representative images of Van Geisson stained ascending aortic segments (E); Scale bar is 100μm. N = 3–6 mice per group. Mean+SEM; P<0.05 (*), P<0.01 (**), P<0.001 (***), P<0.0001 (****), One-way ANOVA with Fisher’s post-hoc tests of least significant differences.
(TIF)

## Abbreviations

Akt: protein kinase B
AngII: angiotensin II
ANOVA: analysis of variance
ApoE: apolipoprotein E
ARB: angiotensin II type 1 receptor blocker
A.U.: arbitrary units
C/EBPδ: CCAAT-enhancer binding protein δ
CK: creatine kinase
DMD: Duchenne muscular dystrophy
EDL: extensor digitorum longus
4E-BP1: eukaryotic translation initiation factor 4E-binding protein 1
GAPDH: glyceraldehyde 3-phosphate dehydrogenase
HDL: high-density lipoprotein
HDLc: HDL-associated cholesterol
HFD: high-fat high-cholesterol diet
LDL: low-density lipoprotein
LGMD2B: limb-girdle muscular dystrophy 2B
MAPK: mitogen-activated protein kinase
MD: muscular dystrophy
MDC1A: α2 laminin-deficient congenital MD type 1A
MM: Myoshi myopathy
Mo: months of age
mTORC1: mammalian target of rapamycin complex 1
NADH: nicotinamide adenine dinucleotide
NO: nitric oxide
NOS: nitric oxide synthase
NT: nitro-tyrosine
PECAM: platelet endothelial cell adhesion molecule-1
PPAR-γ: peroxisome proliferator-activated receptor gamma
rpS6: ribosomal protein S6
S6K1: ribosomal protein S6 kinase beta-1
SEM: standard error of the mean
Ser: serine
SMAD: Mothers against decapentaplegic homolog
TA: tibialis anterior
TC: total cholesterol
TG: triglyceride
TGF-β: transforming growth factor β
