# Supplementary figures and images for: Angiotensin II receptor blocker losartan exacerbates muscle damage and exhibits weak blood pressure-lowering activity in a dysferlin-null model of Limb-Girdle muscular dystrophy type 2B
Source: PLoS One. 2019 Aug 12;14(8):e0220903. doi: 10.1371/journal.pone.0220903 (PMC6690544; doi:10.1371/journal.pone.0220903)

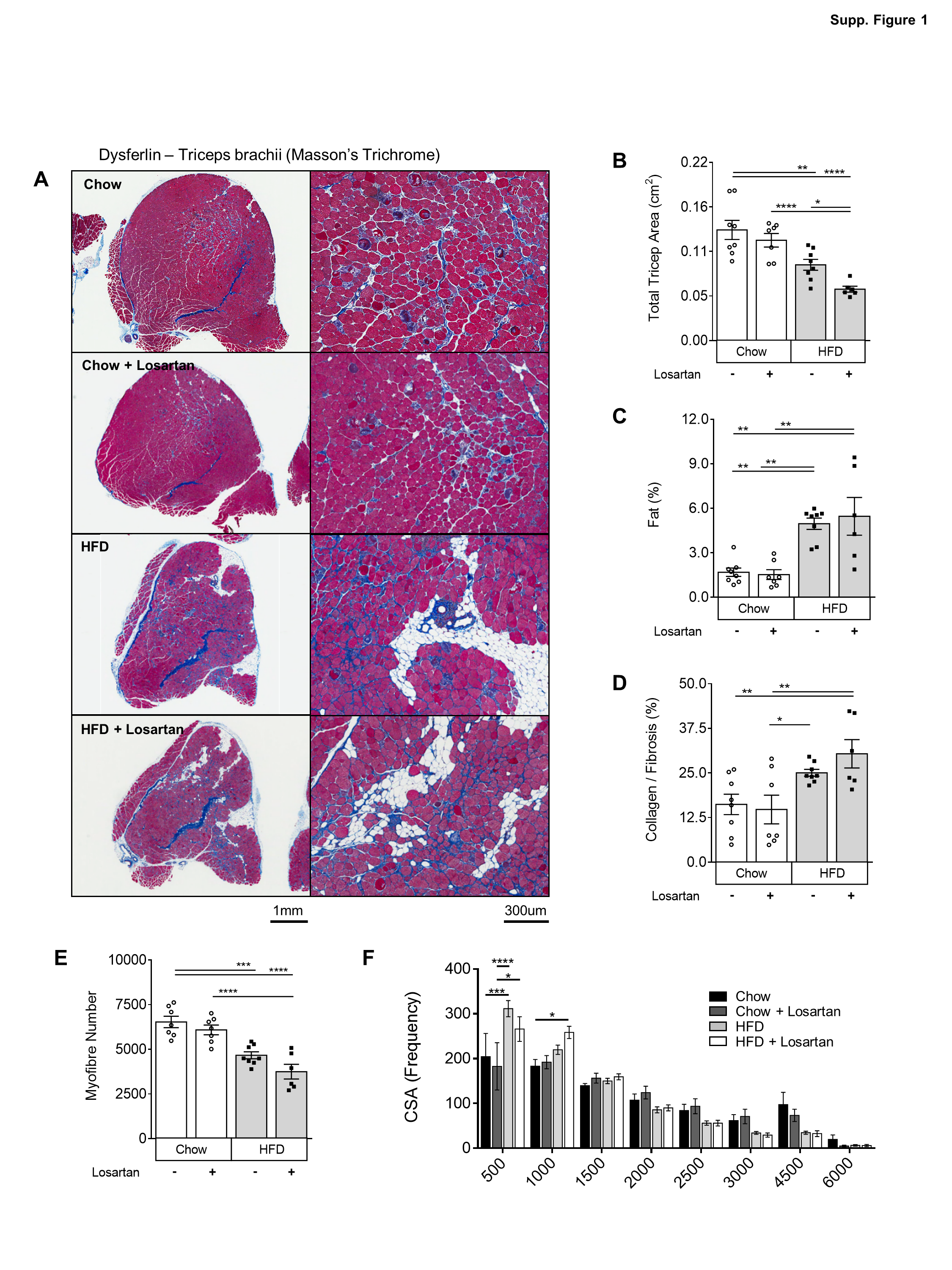

Supplement: S1 Fig — Representative images of triceps brachii from mice after Masson’s trichrome staining (A) and quantification of total triceps brachii area (B), percentage area of fat (C), percentage area of fibrosis/collagen infiltration (D), total myofibre number (E) and cross-sectional area (CSA) frequencies (F). Mean±SEM; P<0.05 (*), P<0.01 (**), P<0.001 (***), P<0.0001 (****), One-way ANOVA with Fisher’s post-hoc tests of least significant differences. Scale bars for 2x and 8x images are 1mm and 300μm, respectively. Muscle tissue (pink); fibrosis (blue); fat/adipocytes (white). Chow (N = 8); Chow losartan (N = 7); HFD (N = 8); HFD losartan (N = 6). (TIF) [file pone.0220903.s001.TIF]

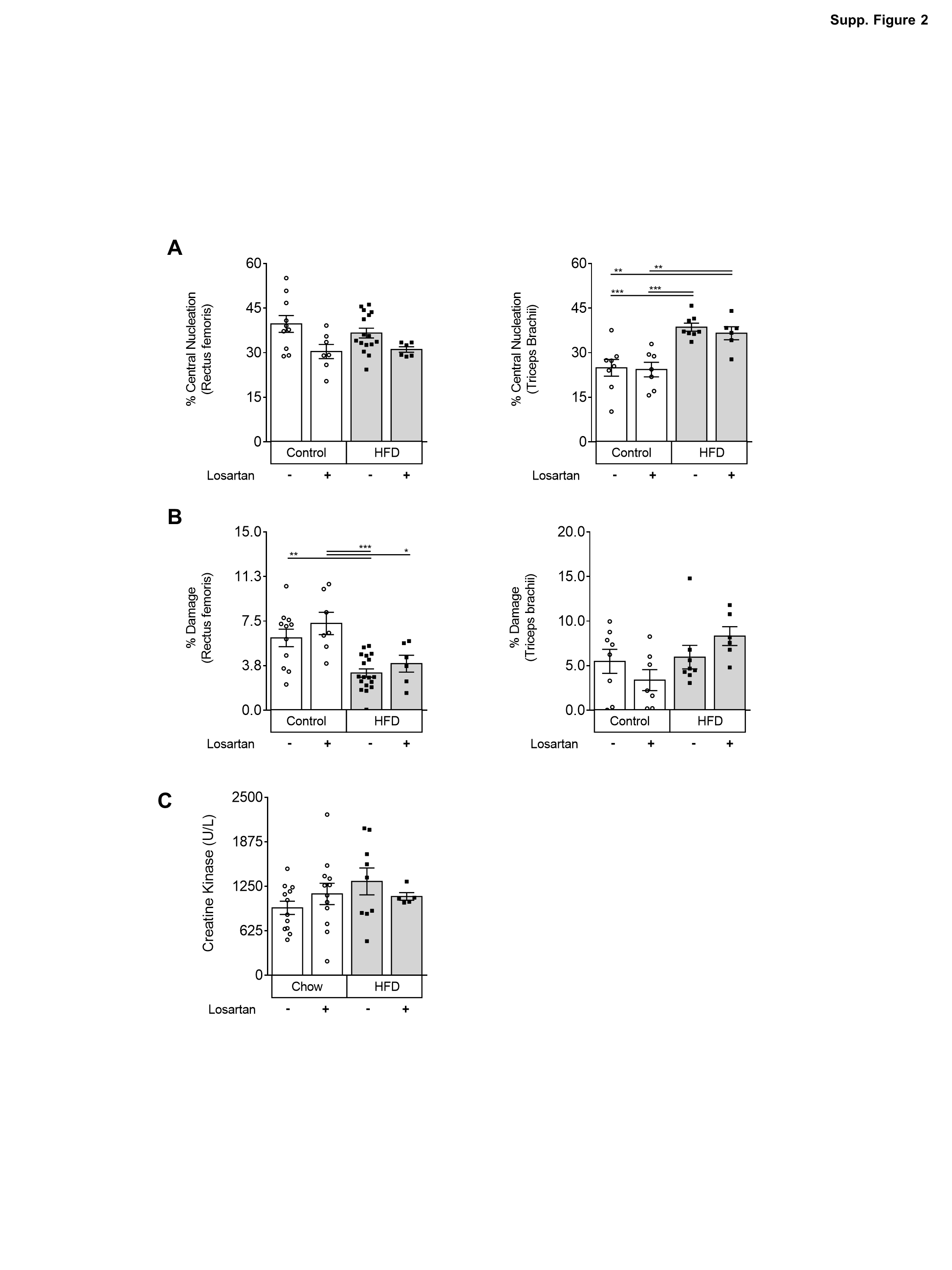

Supplement: S2 Fig — Percentage of centrally nucleated myofibres (A) and the percentage of muscle damage (B) in rectus femoris and triceps brachii muscle groups, and levels of plasma CK (C). Mean±SEM; P<0.05 (*), P<0.01 (**), P<0.001 (***), P<0.0001 (****), One-way ANOVA with Fisher’s post-hoc tests of least significant differences. Chow (N = 12); Chow losartan (N = 12); HFD (N = 9); HFD losartan (N = 5). (TIF) [file pone.0220903.s002.TIF]

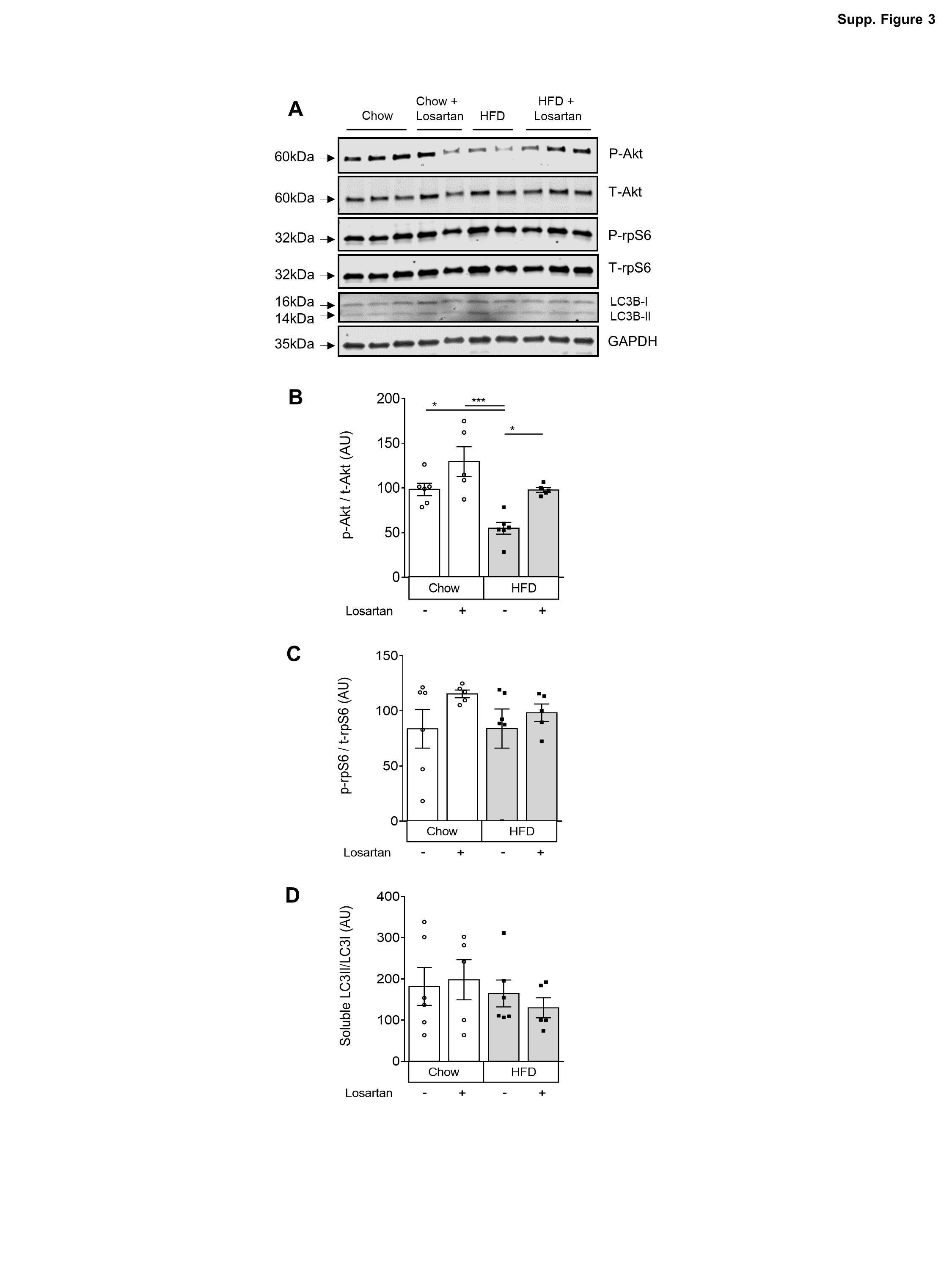

Supplement: S3 Fig — Quantitation of p-AKT(Ser473) standardised to t-AKT (A,B), p-rpS6(Ser235/236) to t-rpS6 (A,C) and ratio of LC3BII/I (A,D). Mean+SEM; P<0.05 (*), P<0.01 (**), P<0.001 (***). One-way ANOVA with Fisher’s post-hoc tests of least significant differences. Y-axes represent arbitrary units (A.U). p-AKT, p-rpS6 and LC3B were cut from the same gel and blotted with respective antibodies, as were t-AKT and t-rpS6. Both total and phosphorylated strips for rpS6 were stripped and blotted for GAPDH to check for loading efficiencies. Full blots were imaged separately and thus have differing exposures. Chow (N = 6); Chow losartan (N = 5); HFD (N = 6); HFD losartan (N = 5). (TIF) [file pone.0220903.s003.TIF]

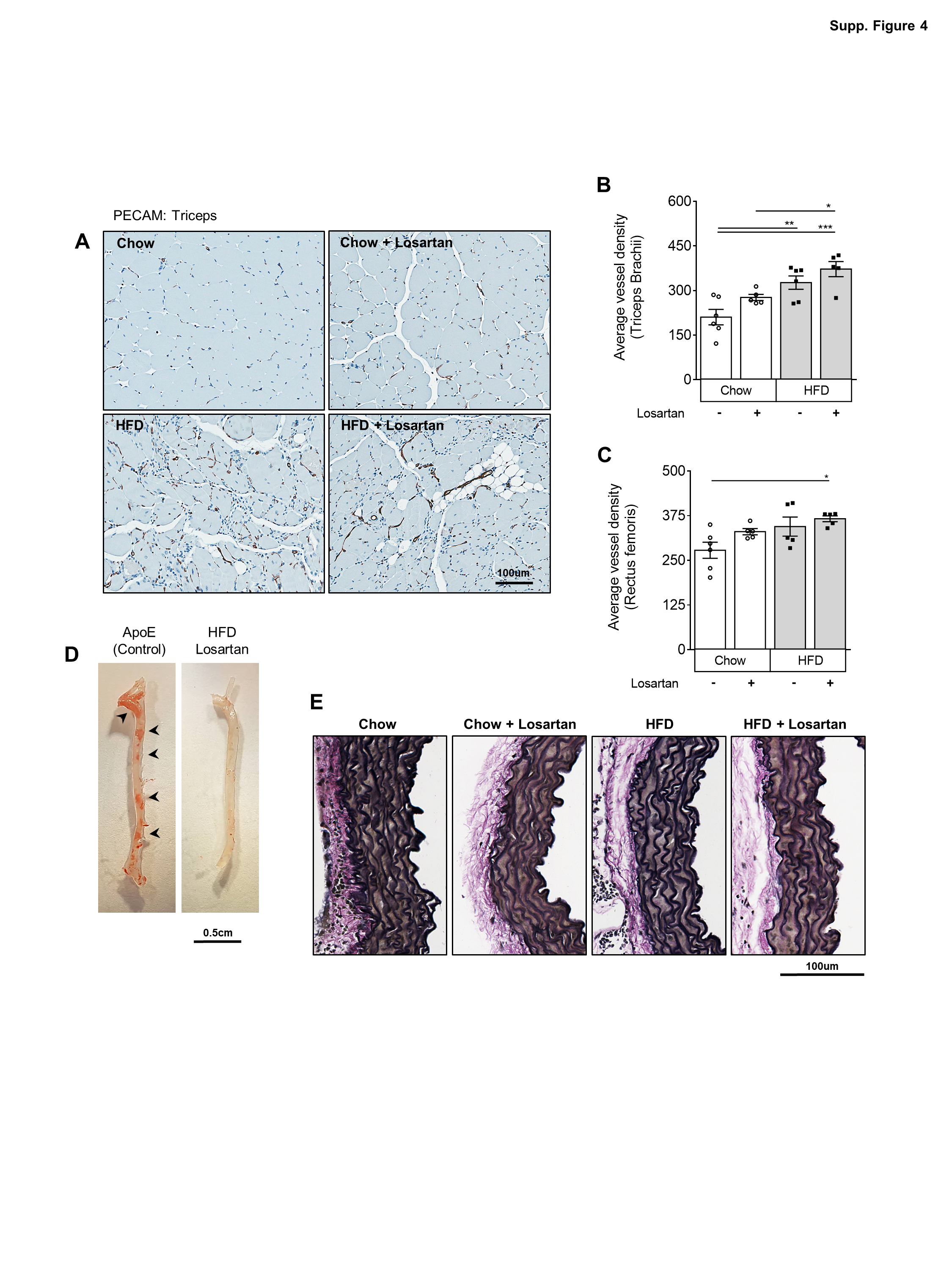

Supplement: S4 Fig — Representative images (triceps brachii only; A) and quantitation of vessel density for triceps brachii rectus femoris (B) and rectus femoris (C); Scale bar is 100μm. Representative images of Sudan IV stained thoracic aorta segments in ApoE (Control) and HFD Losartan treated dysferlin-null mice (D); Scale bar is 0.5cm. Representative images of Van Geisson stained ascending aortic segments (E); Scale bar is 100μm. N = 3–6 mice per group. Mean+SEM; P<0.05 (*), P<0.01 (**), P<0.001 (***), P<0.0001 (****), One-way ANOVA with Fisher’s post-hoc tests of least significant differences. (TIF) [file pone.0220903.s004.TIF]
